# The Use of a Rubber Dam in Abdominal Operations

**Published:** 1900-09-01

**Authors:** 


### THE USE OF A RUBBER DAM IN ABDOMINAL OPERATIONS.

Dr. Fenton B. Turck,[1] of Chicago, with the object of lessening the risk of infection in abdominal surgery, recommends the use of a sheet of indiarubber to cover the skin, so as, on the one hand, to prevent any infection which may reside in the skin doing harm to the exposed intestine, and, on the other, to prevent any extravasation from the intestines which may take place during the operation infecting the skin and thus interfering with aseptic union. The rubber sheet may be made with a hole fitting the seat of operation, and may be provided with flaps which can be tucked in to protect the incision, or it may be stretched over the abdomen like a second skin, and then be divided by the surgeon. This is, of course, merely carrying to a further stage the methods of protection by sterilised towels now in common use. Going a step further, however, he advocates the use of a rubber covering for the wound itself—a covering having suitable holes, the edges of which are strengthened, if necessary, by rubber bands so as to form a sort of elastic collar round the holes. Through these holes the portion of the viscera to be dealt with are drawn; in a case of resection the loop of intestine, in a case of gastro-enterostomy the stomach through one opening and the intestine through another, the bridge between the two being afterwards divided. By this proceeding it is hoped to do away entirely with the necessity for cleaning the peritoneum after the operation is concluded. The idea is doubtless borrowed from the dentists, and if one were provided with a sufficient variety of these sheets, each with its elastic opening, or openings, to fit any case that might arise, it is easy to see that, by the grip given by the elastic edges of these holes, infection of the peritoneum might be prevented with considerable certainty, and with more neatness than by the sponges and towels so commonly used for the purpose. But unless one felt sure that the ring gripped the bowel with sufficient firmness to prevent extravasation the whole affair would probably be but a broken reed. It is, however, intended to be fairly tight, as one of the further advantages claimed for it is the control of hæmorrhage. Another dodge recommended by the same surgeon is the use of thin rubber bags, loosely filled with hot water, and covered or not with gauze, according to the requirements of the case, in place of the sterilised towels or sponges used in certain proceedings to hold back the abdominal contents from the seat of operation. This is recommended with the object of preventing shock, and it is claimed that the utility of the hot bags for this purpose is very great.

[1] New York Med. Rec., Aug. 11.

